# Gracillin shows potent efficacy against colorectal cancer through inhibiting the STAT3 pathway

**DOI:** 10.1111/jcmm.16134

**Published:** 2020-12-01

**Authors:** Lehe Yang, Tianru Zhu, Hua Ye, Yili Shen, Zhiping Li, Luye Chen, Canwei Wang, Xia Chen, Haiyang Zhao, Youqun Xiang, Zhongxiang Xiao, Chengguang Zhao, Jifa Li, Wanle Hu

**Affiliations:** ^1^ The Second Affiliated Hospital and Yuying Children's Hospital Wenzhou Medical University Wenzhou Zhejiang China; ^2^ The First Affiliated Hospital Wenzhou Medical University Wenzhou Zhejiang China; ^3^ Affiliated Yueqing Hospital Wenzhou Medical University Wenzhou Zhejiang China; ^4^ School of Pharmaceutical Sciences Wenzhou Medical University Wenzhou Zhejiang China

**Keywords:** colorectal cancer, gracillin, IL6, inhibitor, STAT3

## Abstract

Colorectal cancer (CRC) accounts for about 10% of all annually diagnosed cancers and cancer‐related deaths worldwide. STAT3 plays a vital role in the occurrence and development of tumours. Gracillin has shown a significant antitumour activity in tumours, but its mechanism remains unknown. The human CRC cell lines HCT116, RKO, and SW480 and immunodeficient mice were used as models to study the effects of gracillin on cell proliferation, migration and apoptosis. These were evaluated by cell viability, colony formation, wound‐healing migration and cell apoptosis assays. Luciferase reporter assay, and immunostaining and western blot analyses were used to explore the specific mechanism through which gracillin exerts its effects. Gracillin significantly reduces viability and migration and stimulates apoptosis in human CRC cells. It also significantly inhibits tumour growth with no apparent physiological toxicity in animal model experiments. Moreover, gracillin is found to inhibit STAT3 phosphorylation and STAT3 target gene products. In addition, gracillin inhibits IL6‐induced nuclear translocation of P‐STAT3. Gracillin shows potent efficacy against CRC by inhibiting the STAT3 pathway. It should be further explored as a unique STAT3 inhibitor for the treatment of CRC.

## 1 INTRODUCTION

Colorectal cancer (CRC) accounts for about 10% of all annually diagnosed cancers and cancer‐related deaths worldwide.^1^ It is the second most common cancer diagnosed in women and the third most common in men.^2^ In the United States, increased screening and the removal of precancerous adenomas resulted in a reduced incidence of CRC. The incidence rate, however, is still growing in several European and Asian countries because of the prevalence of risk factors, such as unhealthy diet, smoking and obesity.^3^ Hence, exploration and application of new biomarkers for diagnosis, prognosis and treatment are still needed to improve tumour management and patients’ survival.

Signal transducer and activator of transcription 3 (STAT3) plays important roles in tumours through its effects on cell proliferation, apoptosis, survival, angiogenesis, metastasis and immunoregulation.^4^, ^5^ Mounting evidence shows that constitutively activated STAT3 contributes to tumour development and progression in most cancers, including breast, prostate, gastric, pancreas, colorectum, cervix, ovary, lung, melanoma and blood cancers.^6^, ^7^, ^8^, ^9^, ^10^ Stimulated by cytokines and growth factors, STAT3 is phosphorylated at tyrosine residue 705 (Tyr705), leading to its dimerization followed by nuclear entry, where it binds to specific elements in the genomic DNA and activates gene transcription.^4^, ^11^, ^12^ Therefore, STAT3 is an attractive target for the development of novel antitumour drugs. Various STAT3 inhibitors have been identified in the past 15 years.^11^, ^13^ However, few have moved into clinical trials, and none has thus far been approved for clinical use.

The genus *Dioscorea* (commonly known as ‘yam’) is made up of about 650 species of plants. *Dioscorea* species contains a variety of substances to promote human health, such as amylose, choline, mucin, steroidal saponins and saponins.^14^ Although a great deal of research has been performed on the different species of *Dioscorea* genus, few have addressed the patient in *D*.* quinqueloba*. Gracillin is a natural steroidal saponin component of *D*.* quinqueloba*.^15^ It has been reported that saponins have antitumour, antimicrobial, antioxidant, pro‐apoptotic and anti‐inflammatory properties.^16^ Recently, gracillin was shown to have significant antitumour activity in many cancers.^17^, ^18^, ^19^ It is reported that gracillin induces apoptosis in HL60 human leukaemic cells via oxidative stress and cell‐cycle arrest at the G1 stage.^17^ Min et al showed that complex II mediates gracillin interference with the mitochondrial function and results in decreased mitochondrial membrane potential, oxidative phosphorylation and ATP synthesis, and increased mitochondrial reactive oxygen species (ROS) production and apoptotic death in cancer cells.^19^ However, its antitumour effect and mechanism of action have not been fully characterized. In this study, cell and animal models were used to analyse the antitumour activity of gracillin in CRC and to elucidate its molecular mechanisms of action. We found that gracillin can inhibit the STAT3 signalling pathway in CRC cells.

## 2 MATERIALS AND METHODS

### 2.1 Antibodies and reagents

Gracillin (HPLC > 98%) was purchased from Herbest (Shanxi, China). The compound used in vitro was dissolved in dimethyl sulphoxide (DMSO). The antibodies against STAT3, STAT1, P‐STAT5, STAT5, P‐STAT4, STAT4 and GAPDH were purchased from Cell Signaling Technology. The antibodies against P‐STAT3, Survivin, VEGF, Mcl‐1, BAX, cleaved caspase3, P‐STAT1 and lamin‐B were purchased from Abcam Co. The horseradish peroxidase (HRP)‐conjugated donkey anti‐rabbit IgG, HRP‐conjugated goat anti‐mouse IgG and the anti‐Bcl‐2 antibody were purchased from Santa Cruz Biotechnology Inc. The Annexin V‐FITC Apoptosis Detection Kit I was obtained from BD Pharmingen. Methylthiazolyldiphenyl‐tetrazolium bromide (MTT) and DMSO were obtained from Sigma‐Aldrich Co. Hoechst 33 258 was purchased from Beyotime Institute of Biotechnology. The Lipofectamine 3000 was purchased from Thermo Fisher Scientific. siRNA was purchased from GenePharma. The Dual‐Luciferase Report Assay Kit was obtained from Promega Biotech Co., Ltd. A Bradford protein‐assay kit, polyvinylidene fluoride membrane, enhanced chemiluminescence kit, acrylamide (30%), Coomassie Brilliant Blue, tetramethylethylenediamine, Tris‐glycine, sodium dodecyl sulphate, pre‐stained protein marker and non‐fat dry milk were obtained from Bio‐Rad Laboratories. The protease phosphatase‐inhibitor mixture was obtained from Applygen Technologies.

### 2.2 Cell culture

Cells of the human CRC cell lines HCT116 (RRID:CVCL_0291), RKO (RRID:CVCL_0504) and SW480 (RRID:CVCL_0546) were obtained from the Shanghai Institute of Biosciences and Cell Resources Center (Chinese Academy of Sciences, Shanghai, China). All the cell lines have been authenticated using STR profiling within the last three years. All experiments were performed with mycoplasma‐free cells. RKO and SW480 cells were maintained in Roswell Park Memorial Institute (RPMI) 1640 medium containing 10% foetal bovine serum (FBS) and antibiotics (Gibco). HCT116 cells were maintained in McCoy's 5A medium supplemented with 10% FBS. All cells were grown in a humidified incubator with a 5% CO_2_ atmosphere.

### 2.3 MTT cytotoxicity assay

Viability of the human CRC cells was evaluated by the MTT assay. Cells (3000‐5000 cells/well) were seeded in 96‐well plates and cultured overnight. After being treated with appropriate drugs for 48 hours, cells were incubated with 20 μL MTT (5 mg/mL) for 4 hours. The medium was then replaced with 150 μL DMSO in each well. The plate was agitated on a plate shaker for 5 minutes and then scanned for spectrophotometric absorbance at 490 nm. The half‐maximal inhibitory concentration (IC_50_) value was calculated using the GraphPad Prism 7 software.

### 2.4 Colony formation assay

Human CRC cells were seeded for colony formation in 6‐well plates at a density of 1000 cells per well and incubated overnight at 37°C in a 5% CO_2_ atmosphere. Different concentrations of gracillin in DMSO (0, 2.5, 5.0 or 10.0 µmol/L) were added. After 7‐10 days (medium exchanged every two days), colonies were washed with PBS and then fixed with 4% paraformaldehyde. After carefully washing three times with PBS, the colonies were stained with crystal violet for 10 minutes and observed under a light microscope.

### 2.5 Wound‐healing assay

Cell migration was evaluated by the wound‐healing assay. RKO cells were plated in 6‐well plates at an appropriate seeding density of 1 × 10^6^ cells/well. When the cells grew to 80%‐90% confluence, the cell monolayers were mechanically scarred with a sterile 200 μL pipette tip along the centre of the well to generate a clean, straight wound area. Different concentrations of gracillin (1.0, 2.5 or 5.0 µmol/L) and 2% FBS were added to the culture media, and the plated were incubated for 24 hours. The scars were observed and measured at 0 hour and 24 hours by light microscopy.

### 2.6 Cell apoptosis assay

Cell apoptosis assay was performed as previously described.^7^ Apoptosis was evaluated using an apoptosis detection kit (BD Biosciences). RKO and HCT116 cells were seeded into 6‐well plates and treated with gracillin (2.5, 5.0 or 10.0 µmol/L) for 36 hours. Cells were washed twice in ice‐cold PBS before being harvested. The harvested cells were double stained according to the instructions of the apoptosis kit. FACSCalibur flow cytometer (BD Biosciences) was used to evaluate cell apoptosis.

### 2.7 Hoechst 33 258 staining assay

Apoptosis in RKO and HCT116 cells was detected by the Hoechst 33 258 assay. The cells were seeded in 6‐well plates and incubated overnight. Gracillin (2.5, 5.0 or 10.0 µmol/L) was added into the wells. After 36 hours, the cells were washed carefully with PBS and fixed with 4% paraformaldehyde for 15 minutes. The samples were then washed twice with PBS. The cells were incubated with Hoechst 33 258 for 20 minutes, washed with PBS and observed by fluorescence microscopy.

### 2.8 Western blot analysis

Human CRC cells were seeded in 6‐well plates (500 000/well) and incubated overnight at 37°C in an atmosphere of 5% CO_2_. Different concentrations of gracillin were added into the wells. Total protein was extracted and separated by SDS‐PAGE. Following electrophoresis, the proteins in the gel were transferred to polyvinylidene difluoride membranes. After blocking for 1 hour with 5% non‐fat milk, the membranes were incubated overnight at 4°C with specific primary antibodies. The next day, the blots were incubated with relevant secondary antibodies. Visualization of the target proteins was enhanced with Electrochemiluminescence (ECL) substrate reagents.

### 2.9 Molecular docking of gracillin to the binding spots of STAT3

Molecular docking was performed with AutoDock Vina 1.0.2.^20^ The crystal structure of the STAT3/DNA complex (PDB code 1BG1) derived from Protein Data Bank was used for our docking study. The input files of ligand and receptor were prepared using Graphical User Interface program AutoDock Tools 1.5.6^21^ (The Scripps Research Institute). During the docking, the receptor was considered as rigid whereas the ligand was flexible.

### 2.10 STAT3 luciferase report assay

The STAT3 luciferase reporter plasmid (pGLSTAT3‐Luc) was used to detect STAT3 activation, as was previously described.^8^ Briefly, HCT116 cells were seeded in 24‐well plates and incubated for 24 hours before transfection. The cells were co‐transfected with pGLSTAT3‐Luc and pRL‐TK (a plasmid encoding Renilla luciferase) using Lipofectamine 3000 (Invitrogen) for 6 hours. Finally, the cells were treated with different concentrations of gracillin for 12 hours. Luciferase activity was assessed by SpectraMax ID3 (Molecular Devices). The inhibition of STAT3 activity by gracillin was calculated as the ratio between the value of firefly and Renilla luciferase activity. Each experiment was carried out in triplicate in three independent experiments.

### 2.11 Transient transfection of small interfering RNA (siRNA)

GenePharma (Shanghai, China) designed and synthesized the siRNA against STAT3 (si‐STAT3) and a negative control siRNA (si‐NC). CRC cells were transiently transfected according to the manufacturers’ instructions. Sequences of STAT3 siRNAs were as follows: si#1: STAT3‐Homo398 5′‐CCACUUUGGUGUUUCAUAATT‐3′; si#2: STAT3‐Homo‐1070 5′‐CCCG UCAACAAAUUAAGAATT‐3′; si#3: STAT3‐Homo‐978 5′‐GCAACAGAUUGCCUGC AUUTT‐3′.

### 2.12 Immunofluorescent staining

Briefly, HCT116 cells were seeded onto glass coverslips. When they reached 70% confluence, they were washed once in PBS and then fixed with 4% paraformaldehyde for 15 minutes at room temperature. Coverslips were washed three times in excess of PBS, permeabilized with −20°C 100% methanol for 30 minutes and then blocked in 1% bovine serum albumin (BSA) for 30 minutes. The cell preparations were incubated overnight in the dark at 4°C with a specific primary anti‐P‐STAT3 antibody (1:100 in 3% BSA). On the following day, the cell preparations were incubated at room temperature for 1 hour with PE‐labelled goat anti‐rabbit IgG antibody (1:200). After that, the nuclei were stained with DAPI. The images were acquired by confocal microscopy (Leica) using a × 63 oil lens.

### 2.13 Cytoplasmic and nuclear protein extraction

The cytoplasmic and nuclear proteins of HCT116 cells were isolated by NE‐PER Nuclear & Cytoplasmic extraction kit (Thermo Fisher Scientific). Different concentrations of gracillin were added to the plates. Cells were stimulated with IL6 for 20 minutes before being harvested according to the manufacturer's protocol. Protein expression in the cytoplasmic and nuclear fractions was detected using immunoblot analysis.

### 2.14 Animal experiments

All animal experiments were conducted following the institutional ethics and safety guidelines (Institutional Animal Welfare and Ethics Committee, Wenzhou Medical University, China). Five‐week‐old athymic female BALB/c nude mice (n = 18; 18‐20 g) were used in the experiments. The mice were fed and treated according to the Wenzhou Medical University's Policy on the Care and Use of Laboratory Animals. HCT116 cells were harvested and then transplanted subcutaneously (5 × 10^6^ cells in 100 μL of PBS) on the right flank of the mice. The mice were randomly divided into three groups, which were injected intraperitoneal every other day with gracillin (1 mg/kg or 2 mg/kg) or PBS (control) till the tumours’ volume reached ~100 mm^3^. The tumours volume (V) was calculated using the following formula: V = 0.5 × A×B × B, in which A is the length and B is the width. Finally, mice were killed, and their hearts, livers, kidneys and lungs were fixed immediately and paraffin‐embedded. The tumours were photographed, weighed, measured and preserved at −80°C till they were used for other experiments.

### 2.15 Haematoxylin and eosin (H&E) staining

Tissue slices from the hearts, lungs, livers and kidneys were fixed in 4% paraformaldehyde and embedded in paraffin. They were then sectioned (5μm), deparaffinized, rehydrated and then stained with eosin and haematoxylin. Images were acquired with a light microscope.

### 2.16 Statistical analysis

Data are expressed as mean ± SD of three independent experiments. The different groups were compared by Student's *t* test or one‐way analysis of variance using the GraphPad Pro 7.0 software (GraphPad). Differences were considered significant when *P* < .05.

## 3 RESULTS

### 3.1 Gracillin inhibits proliferation and migration of human CRC cells

**FIGURE 1:**
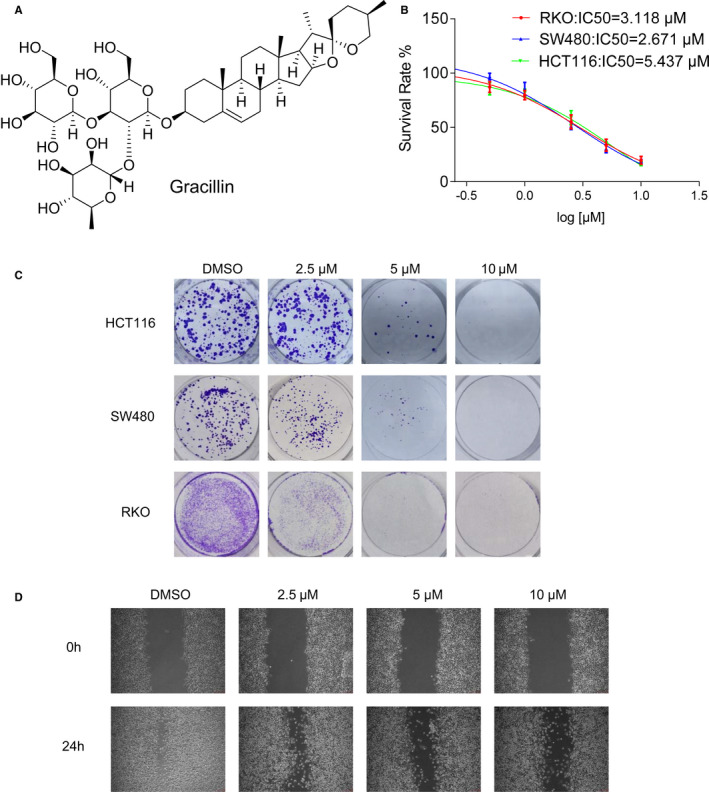
Gracillin inhibits proliferation and migration of human CRC cells. A, Chemical structure of gracillin. B, RKO, HCT116 and SW480 cells treated with increasing concentrations of gracillin for 48 h. Cells’ viability was evaluated by the MTT assay, and the half‐maximal inhibitory concentration (IC_50_) was calculated using the GraphPad Prism software. Data are presented as means of three experiments, and error bars represent SD. C, HCT116, SW480 and RKO cells were incubated with different concentrations of gracillin (0, 2.5, 5.0 or 10.0 μmol/L) for 24 h. Colonies were fixed, and micrographs were acquired. D, RKO cell cultures, grown to 80%‐90% confluence, were scratched to form 200 µm‐wide wounds. Cells were then treated with gracillin at different concentrations (0, 1.0, 2.5 or 5.0 μmol/L) for 24 h. Micrographs were acquired at 0 h and 24 h after treatment with gracillin

We used the MTT and colony formation assays to identify gracillin's inhibitory effect on the human CRC cells (Figure 1A). As shown in Figure 1B, gracillin significantly inhibits the viability of RKO, SW480 and HCT116 cells, with the IC_50_ values being 3.118, 2.671 and 5.473 µmol/L, respectively. However, gracillin shows low toxicity on LO2 cells compare with cancer cells (Figure S1A). Furthermore, we observed a significant inhibitory effect on human CRC cells’ colony formation in a dose‐dependent manner (Figure 1C). These results suggest that gracillin has a considerable inhibitory effect on human CRC cells’ proliferation in vitro. We also performed a wound‐healing assay to investigate the effect of gracillin on the migration ability of human CRC cells. As shown in Figure 1D, the migration rates in the gracillin‐treated groups were significantly lower than the migration rate in the control group. These results strongly suggest that gracillin inhibits the proliferation and migration of human CRC cells.

### 3.2 Gracillin induces apoptosis in human CRC cells

**FIGURE 2:**
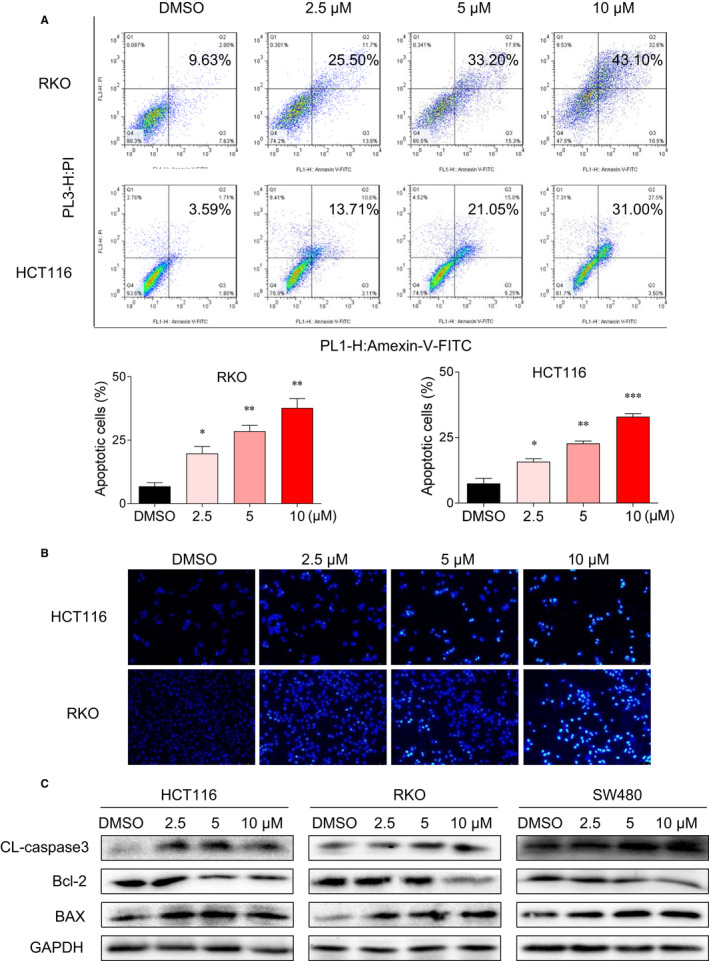
Gracillin induces apoptosis in human CRC cells. A, RKO and HCT116 cells were treated with gracillin at different concentrations (0, 2.5, 5.0 or 10.0 μmol/L) for 36 h. Cells were harvest and then stained with Annexin V‐FITC and PI, before being analysed by flow cytometry. Data are presented as means of three experiments, and error bars represent SD (*P < .05, **P < .01, ***P < .001). B, HCT116 and RKO cells were treated with different concentrations of gracillin (0, 2.5, 5.0 or 10.0 μmol/L) for 36 h. Cells were stained with Hoechst 33 258, and their apoptotic morphological characteristics were analysed. C, HCT116, RKO and SW480 cells were treated with gracillin at different concentrations (0, 2.5, 5.0 or 10.0 μmol/L) for 36 h. The expression levels of Bcl‐2, BAX, cleaved caspase‐3 and GAPDH were evaluated by Western blot analysis

To assess the apoptosis‐induction ability of gracillin, RKO and HCT116 cells were treated with increasing concentrations of gracillin for 36 hours. Samples were then stained with Annexin V‐FITC and PI and evaluated by flow cytometry. Results show that gracillin stimulates apoptosis in human CRC cells in a dose‐dependent manner (Figure 2A). Furthermore, HCT116 and RKO cells were treated with gracillin for 36 hours and then stained with Hoechst 33 258 to assess the morphological features of the apoptotic cells. The percentage of gracillin‐induced apoptotic cells was higher than that in the untreated control group (Figure 2B). Moreover, gracillin could enhance the expression levels of cleaved caspase‐3 and BAX and decrease the expression level of Bcl‐2, confirming its pro‐apoptotic effect on human CRC cells (Figure 2C). Collectively, these results indicate that gracillin induces apoptosis in human CRC cells.

### 3.3 Gracillin inhibits the STAT3 signalling pathway in human CRC cells

**FIGURE 3:**
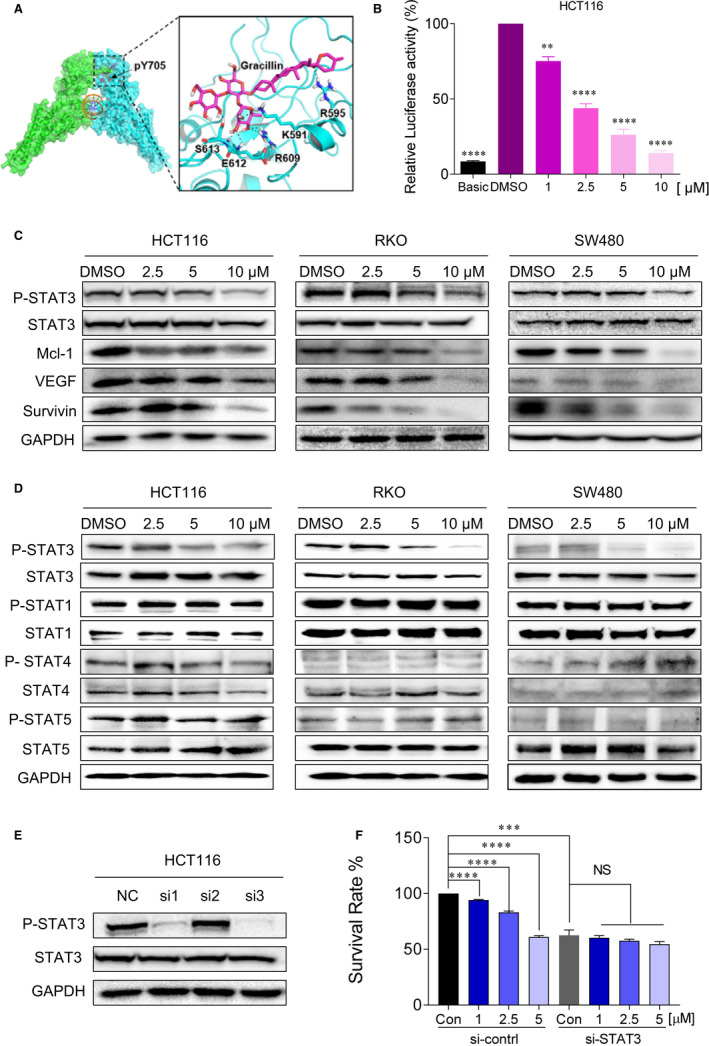
Gracillin inhibits the STAT3 signalling pathway in human CRC cells. A, Molecular docking of gracillin to the STAT3 binding spots. B, HCT116 cells were transfected with luciferase reporter gene plasmid and treated with gracillin for 12 h. The results were normalized to the Renilla luciferase activity. **P < .01; ****P < .0001. C, HCT116, RKO and SW480 cells were treated with different concentrations of gracillin (0, 2.5, 5.0 or 10.0 μmol/L) for 12 h. Expression levels of P‐STAT3, STAT3, Mcl‐1, GAPDH, VEGF and Survivin were evaluated by the Western blot assays. D, HCT116, RKO and SW480 cells were treated with different concentrations of gracillin (0, 2.5, 5.0 or 10.0 μmol/L) for 12 h. Expression levels of P‐STAT3, STAT3, P‐STAT1, STAT1, P‐STAT4, STAT4 and P‐STAT5, STAT5 and GAPDH were evaluated by the Western blot assays. E, HCT116 cells were transfected with si‐NC or si‐STAT3 (si#1, si#2 or si#3) for 24 h. Expression levels of P‐STAT3, STAT3 and GAPDH were detected by the Western blot assay. F, HCT116 cells were transfected with si‐NC or si‐STAT3 (si#3) for 24 h and then treated with gracillin at different concentrations (0, 1.0, 2.5 or 5.0 μmol/L) for 48 h. Cell proliferation was evaluated by the MTT assay (***P < .001, ****P < .0001)

The docking result (Figure 3A) reveals that gracillin can occupy the pY705‐binding site and form eight hydrogen bond with residue R595, K591, R609, R612, E612 and S613, respectively. This result suggests that gracillin might disrupt the native phosphorylated Tyrosine 705 peptide binding and possibly inhibit STAT3 phosphorylation. To further confirm the STAT3 inhibitory effect of gracillin, we detected STAT3 constitutive activation in HCT116 and RKO cells by using the luciferase reporter assay. We found that gracillin significantly inhibits P‐STAT3 activation in a dose‐dependent manner (Figure 3B and Figure S2A). Western blot assay was employed to determine the expression of the STAT3 signalling pathway‐associated proteins. In agreement with the data obtained using the luciferase reporter assay, the P‐STAT3 level and the levels of STAT3‐related proteins (Mcl‐1, VEGF and Survivin) were down‐regulated by gracillin in human CRC cells in a dose‐dependent manner (Figure 3C). To investigate the selective inhibitory effect of gracillin, we evaluated the effect of gracillin on the activation of P‐STAT3, P‐STAT1, P‐STAT4 and P‐STAT5 in CRC cells. As shown in Figure 3D and Figure S3, gracillin did not reduce the protein levels of phosphorylated STAT1, STAT4 and STAT5. HCT116 cells were transfected with three different sequences of si‐STAT3 (si#1, si#2 and si#3). Among them, si#3 showed the highest STAT3 inhibitory activity (Figure 3E). It was therefore selected for subsequent transient transfection assays. To confirm the ability of gracillin to inhibit cell proliferation and to test whether this effect was STAT3‐dependent, we examined the inhibitory effect of gracillin on the proliferation of HCT116 cells, which were depleted or not of STAT3 (Figure 3F). Gracillin did not affect the siSTAT3‐transfected cells. In sharp contrast, gracillin was able to inhibit cell proliferation in HCT116 cells transfected with a control siRNA (and therefore expressing STAT3). In brief, these results suggest that gracillin exerts its inhibitory effect through the STAT3 signalling pathway.

### 3.4 Gracillin inhibits nuclear translocation of STAT3 in human CRC cells

**FIGURE 4:**
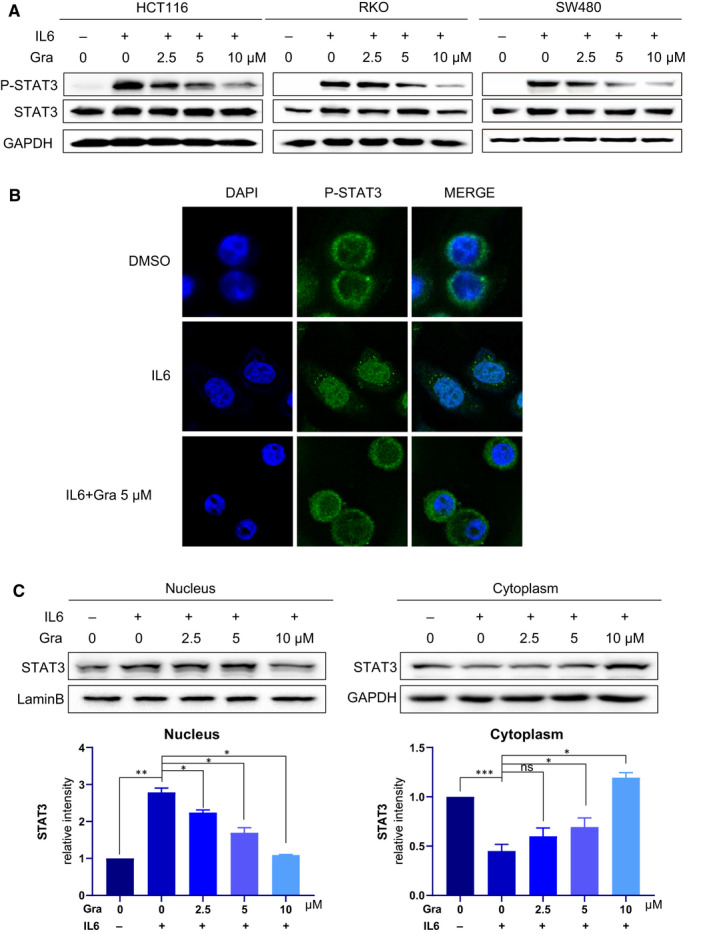
Gracillin inhibits nuclear translocation of P‐STAT3 in human CRC cells. A, Cells were pre‐treated with the indicated concentrations of gracillin for 12 h and then stimulated with IL‐6 (25 ng/mL) for 30 min. Cell extracts were prepared and subjected to the Western blot analysis using the indicated antibodies. B, HCT116 cells were treated with 5 μmol/L gracillin for 12 h. Cells were exposed to IL6 (25 ng/mL) for 30 min at the end of the 12‐h incubation period. We used immunostaining to assess the localization of P‐STAT3 (green) and 4,6‐diamidino‐2‐phenylindole (DAPI; blue) in HCT116 cells. C, The expression of P‐STAT3 was induced by IL6 (25 ng/mL), and this effect was reversed by gracillin in a dose‐dependent manner. The expression levels of STAT3 in the nuclear and cytosolic fractions were determined by Western blot analysis. Samples were measured in triplicate, and experiments were independently repeated three times (*P < .05, **P < .01, ***P < .001)

We first investigated whether the STAT3 activation stimulating by IL6 could be disturbed through the treatment of gracillin. As shown in Figure 4A, gracillin can inhibit IL6‐induced STAT3 activation in three CRC cell lines. We next determined whether gracillin treatment affected STAT3 nuclear translocation in HCT116 cells. As expected, exposure of HCT116 cells to 25 ng/mL IL6 for 30 minutes resulted in increased translocation of P‐STAT3 (green fluorescence) from the cytoplasm to the nucleus (blue fluorescence, DAPI stained). Gracillin was found to inhibit this translocation (Figure 4B,C). These results further confirm the inhibitory effect of gracillin on IL6‐induced nuclear translocation of P‐STAT3. These results strongly suggest that the antitumour effect of gracillin is mediated by blocking the STAT3 signalling pathway.

### 3.5 Gracillin inhibits CRC tumour growth in a xenograft model

**FIGURE 5:**
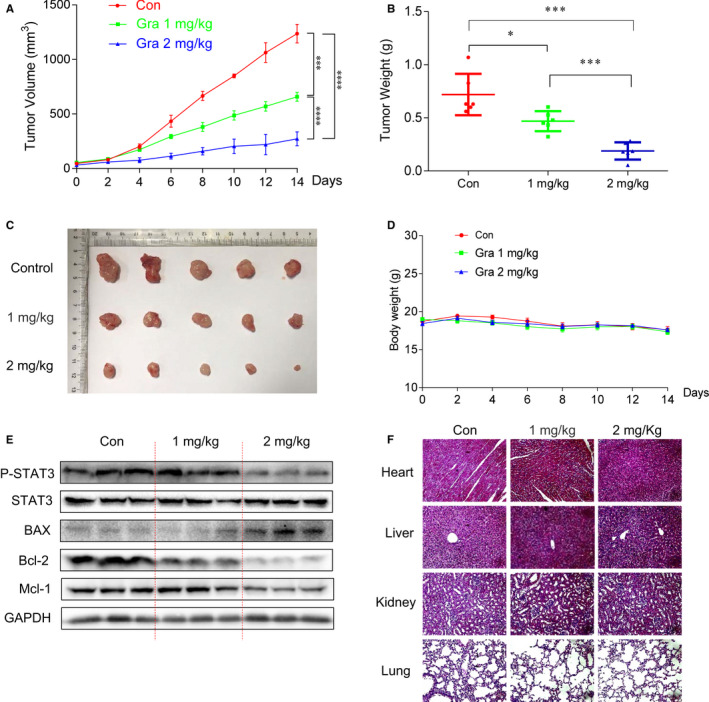
Gracillin inhibits CRC tumour growth in a xenograft model. A, BALB/c nude mice were administered gracillin intraperitoneally (1 or 2 mg/kg) every other day. Tumour volume (V) was calculated based on its length (A) and width (B), according to the following formula: V = 0.5 × A × B × B. (B) Tumour weight (n = 6). C, Representative images of the solid tumour in the control and gracillin‐treated groups. D, Mice total bodyweight. E, Proteins extracted from tumour tissues were evaluated by the Western blot assay. F, Tissue samples derived from the hearts, livers, kidneys and lungs of mice from the three groups and stained with H&E. All images were captured using light microscopy. Data are presented as means of three experiments, and error bars represent SD (*P < .05, ***P < .001, ****P < .0001)

To further identify the antitumour effect of gracillin, we established xenograft models. When the tumours were visible, the mice were administered with a corresponding concentration of gracillin (1 mg/kg or 2 mg/kg) every other day. Results clearly show that the volume and weight of the tumours were lower in the gracillin‐treated mice when compared to the control group (Figure 5A,B). This inhibitory effect was in a clear dose‐dependent manner. Gracillin led to a supra‐additive effect that resulted in a decrease in the growth of the xenograft tumours (Figure 5C) and the maintenance of a stable bodyweight of the mice (Figure 5D). In addition, we analysed the expression of STAT3‐related proteins in tumour tissues by immunoblot. As shown in Figure 5E, treatment with gracillin inhibited the phosphorylation of STAT3 and the expression of important proteins in the STAT3 signalling pathway. The apoptotic effect of gracillin on CRC in vivo was further demonstrated by using the Western blot assay. The results show that the levels of BAX and cleaved caspase‐3 have increased and that of Bcl‐2 has decreased in tumours of the gracillin‐treated mice. Moreover, gracillin treatment caused no observable toxicity to the heart, liver, kidneys or lungs as compared with that in the vehicle group (Figure 5F), thus demonstrating its excellent safety profile.

## 4 DISCUSSION

The recently discovered cancer‐promoting functions of STAT3 further emphasize the importance of targeting STAT3 activities related to the mitochondria, epigenetic regulation, cancer stem cells, pre‐metastatic niches and more.^22^, ^23^ The STAT3‐Fam3a axis has been reported to promote the progression of the muscle stem cell myogenic lineage by enhancing the mitochondrial respiration.^24^ STAT3 can mediate the epigenetic silencing of tumour suppressor genes (TSG). Gambi and his colleagues confirmed that oncogene‐driven constitutive STAT3 acetylation was responsible for the silencing of TSG. Furthermore, they showed that the Sin3a transcriptional repressor protein is an obligatory partner of STAT3 in promoting genes repression, revealing the mechanisms involved in STAT3‐mediated transcriptional regulation.^25^ Agarwal et al found that G‐CSF promotes tumorigenicity and metastasis of neuroblastoma through STAT3‐dependent cancer stem cell activation.^26^ Liu et al described a novel function of STAT3 in regulating the pH by interacting with the vacuolar H+‐ATPase on lysosomes. This discovery proposes a new anti‐cancer strategy by limiting this function.^27^ It was also shown that the inhibition of the STAT3 pathway by a small‐molecule inhibitor or siRNA significantly enhanced the stimulator of interferon genes (STING) signalling, which was induced by the STING agonist c‐diAM (PS)2. Important to note that these STAT3 inhibitors did not induce the STING signalling by themselves.^28^ All these recent discoveries point to new directions for targeting STAT3 in cancer treatment. Regrettably, thus far, no STAT3 inhibitor has been approved for cancer therapy. To develop a more successful treatment for cancer, we have done much work on finding an effective STAT3 inhibitor.^29^, ^30^

Agents derived from natural sources have attracted much attention because of their safety, efficacy and immediate availability.^4^ They are the best sources for drugs and can lead to novel drug discovery.^31^ Some natural products and derivatives have been found to possess STAT3 inhibitory effect, such as rhein,^7^, ^8^ alantolactone^32^ and others. Here, we identified a new STAT3 inhibitor. In this study, we used CRC cells and animal models to examine the antitumour effects of gracillin and its downstream regulatory mechanism. The results show that gracillin significantly inhibits the proliferation and migration (Figure 1) and induces apoptosis in CRC cells (Figure 2). In animal experiments, we found that gracillin could significantly inhibit tumour growth without any apparent physiological toxicity (Figure 5).

Moreover, gracillin was found to inhibit STAT3 phosphorylation in CRC cells, as evaluated by the STAT3‐dependent dual‐luciferase reporter system. Gracillin inhibits not only STAT3 but also regulates the expression of STAT3 target gene products, including Bcl‐2, Mcl‐1, Survivin and VEGF (Figure 3). Moreover, gracillin did not affect siSTAT3 cells, indicating that the ability of gracillin to inhibit cell proliferation depends on STAT3. In addition, we found, using immunofluorescence staining, that gracillin inhibits the IL6‐induced nuclear translocation of P‐STAT3 (Figure 4). Our data demonstrate that gracillin shows potent efficacy against CRC by inhibiting the STAT3 pathway. Moreover, we have shown that gracillin has few side effects on the mice at the therapeutic concentration used in this study. Reciprocal cross talk between the STAT3 and EGFR pathways is a key molecular mechanism leading to resistance in cancer cells.^5^, ^32^, ^33^ We plan to investigate the possible combination of gracillin with other clinical cancer drugs to improve its antitumour potency and verify its effect on CRC.

## 5 CONCLUSIONS

Our findings provide a convincing proof that gracillin possesses a potent anti‐CRC activity by inhibiting the STAT3 pathway. Gracillin should be further explored as a unique STAT3 inhibitor for the treatment of CRC.

## CONFLICT OF INTEREST

The authors declare that they have no known competing financial interests or personal relationships that could have appeared to influence the work reported in this paper.

## AUTHOR CONTRIBUTIONS

**Lehe Yang:** Writing‐original draft (equal). **Tianru Zhu:** Writing‐original draft (equal). **Hua Ye:** Data curation (equal). **Yili Shen:** Investigation (equal); Methodology (equal). **Zhiping Li:** Methodology (equal). **Luye Chen:** Methodology (equal). **Canwei Wang:** Methodology (equal). **Xia Chen:** Software (equal). **Haiyang Zhao:** Investigation (equal). **Youqun Xiang:** Data curation (equal). **Zhongxiang Xiao:** Conceptualization (equal); Supervision (equal); Writing‐review & editing (equal). **Chengguang Zhao:** Resources (equal); Supervision (equal). **Jifa Li:** Conceptualization (equal); Supervision (equal). **Wanle Hu:** Writing‐review & editing (equal).

## Supporting information

Fig S1‐S3

## Data Availability

The data that support the findings of this study are available from the corresponding author upon reasonable request.
