# Supplementary figures and images for: Gracillin shows potent efficacy against colorectal cancer through inhibiting the STAT3 pathway
Source: J Cell Mol Med. 2020 Dec 1;25(2):801–12. doi: 10.1111/jcmm.16134 (PMC7812262; doi:10.1111/jcmm.16134)

Figure S1

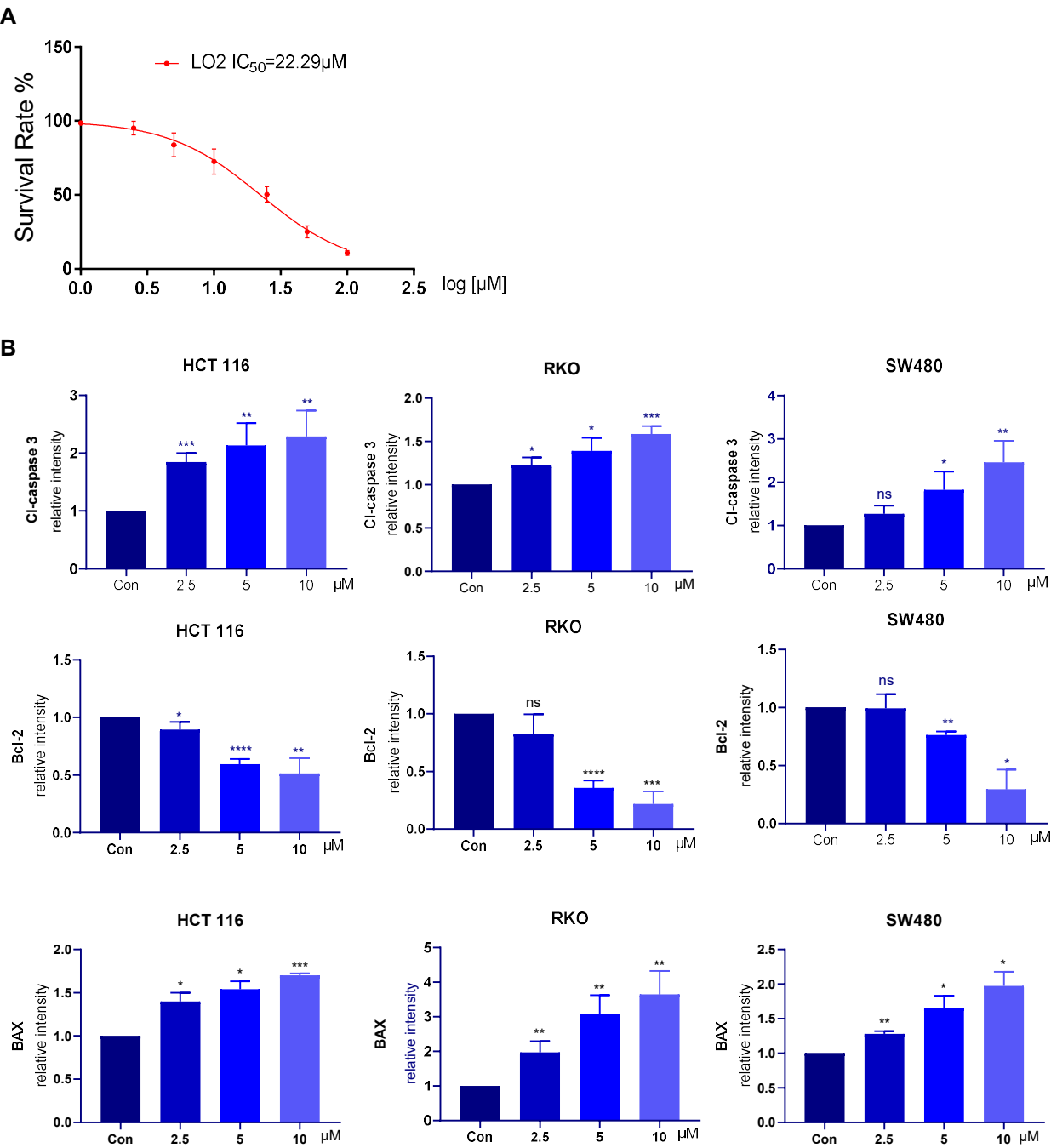

Figure S2

A

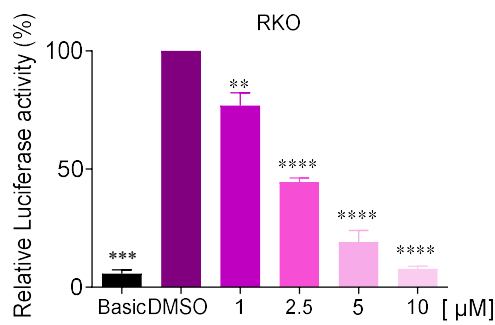

B

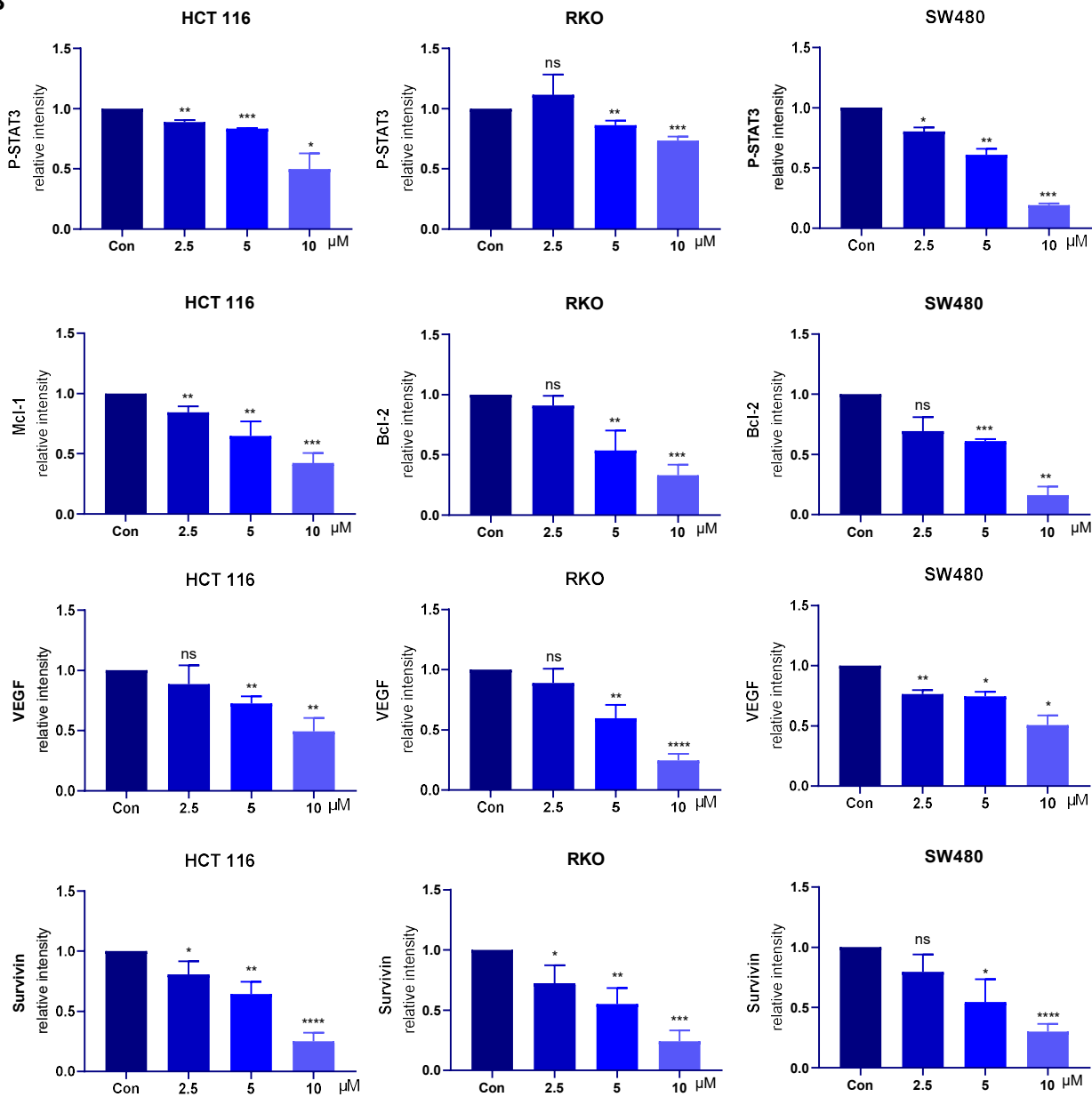

Figure S3

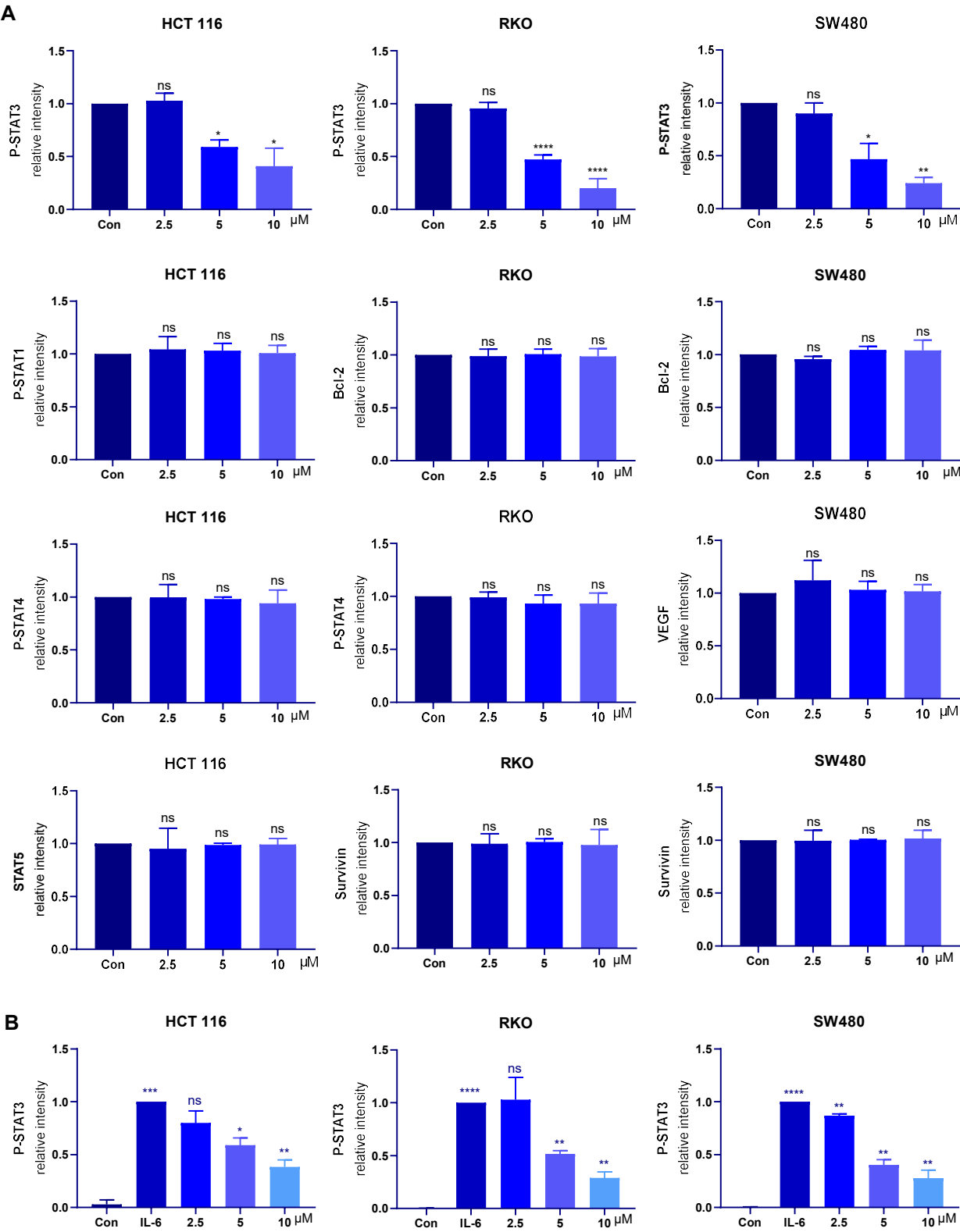

Supplement: Supplementary file 1 — Fig S1‐S3 [file JCMM-25-801-s001.pdf]
